# Intensity modulated and three-dimensional conformal radiation therapy plans for oropharyngeal cancer: a comparison of their sensitivity to set-up errors and uncertainties

**DOI:** 10.3390/curroncol13020005

**Published:** 2006-04

**Authors:** N. Ploquin, H. Lau, P. Dunscombe

**Affiliations:** * Tom Baker Cancer Centre, Department of Medical Physics, and University of Calgary, Department of Physics and Astronomy, Calgary, Alberta; † Tom Baker Cancer Centre, Department of Radiation Oncology, Calgary, Alberta; ‡ University of Calgary, Department of Oncology, Calgary, Alberta

**Keywords:** Equivalent uniform dose, set-up error, head-and-neck cancer, imrt, 3D-crt

## Abstract

We compared the effect of set-up error and uncertainty on two radiation therapy treatment plans for head and neck cancer: one using intensity modulated radiation therapy (imrt) and one using conventional three-dimensional conformal radiation therapy (3D-crt). We used a Pinnacle3 (Philips Medical Systems, Markham, Ontario) system to create the two treatment plans (7-beam imrt and 5-beam 3D-crt) for the same volumetric data set, based on the objectives and constraints defined in the Radiation Therapy Oncology Group H-0022 protocol. In both plans, the dose–volume constraints for the targets and the organs at risk (oars) were met as closely as the beam geometries would allow. Monte Carlo–based simulations of set-up error and uncertainty were performed in three orthogonal directions for 840 simulated “courses of treatment” for each plan. A systematic error (chosen from distributions characterized by standard deviations ranging from 0 mm to 6 mm) and random uncertainties (2 mm standard deviation) were incorporated. We used a probability approach to compare the sensitivities of the imrt and the 3D-crt plans to set-up error and uncertainty in terms of equivalent uniform dose (eud) to targets and oars.

Based on the eud analysis, the targets and oars showed considerably greater sensitivity to set-up error with the imrt plan than with the 3D-crt plan. For the imrt plan, target euds were reduced by 4%, 7.5%, and 10% for 2-mm, 4-mm, and 6-mm set-up errors respectively. However, even with set-up error, the mandible, spinal cord, and parotid euds always remained lower with the imrt plan than with the 3D-crt plan.

We conclude that, when quantified by eud, imrt-plan doses to oars and targets are more sensitive to set-up error than are 3D-crt-plan doses. However, as judged by the differences between target and oar doses, imrt retains its superiority over 3D-crt, even in the presence of set-up error.

## 1. INTRODUCTION

Treatment plan development and evaluation in head and neck cancers are a challenge—in part because of the large number of radiosensitive normal structures in close proximity to the targets. The Radiation Therapy Oncology Group (rtog) designed a complex protocol for cancer of the oropharynx (H-0022) 1 with the main goal of exploring the feasibility and value of delivering adequate target doses while sparing the major salivary glands.

Intensity modulated radiation therapy (imrt) is available in many cancer centres and is becoming a commonly used treatment procedure. In treatment-plan generation, the dose distribution using imrt has been shown to be superior to that using three-dimensional conformal radiation therapy (3D-crt) 2. However, whether imrt retains its superiority in the actual clinical situation, where set-up error and uncertainty are features of every treatment course, has not yet been established.

In accord with reports 50 3 and 62 4 from the International Commission on Radiation Units and Measurements, margins are added to some of the volumes of interest during the treatment planning process to account for set-up error and uncertainty. For example, to account for geometric uncertainties and organ motion, the clinical target volume (ctv), which encompasses the primary tumour and subclinical microscopic disease, is extended by a margin to form the planning target volume (ptv). For head and neck cancer, the expansion of the ctv to a ptv accommodates set-up error and geometric uncertainties, because organ motion and rotation are not significant for these sites 5.

Set-up error and uncertainty in treatment plan evaluation are of considerable current interest 6–9, and they are particularly relevant when highly conformal plans are being considered. When treatment plans are being created and optimized, the evaluation process is almost always based on static volumes. Set-up uncertainty is taken into consideration only through generic margins on selected structures. Based on static distributions, imrt is considered by many to be a superior technique 2. In the presence of set-up uncertainty, the superiority of an imrt plan may well be compromised; however, the degree of that uncertainty is typically unknown.

To avoid ambiguity, we use the term “error” when discussing systematic effects that influence an entire course of treatment and the term “uncertainty” when discussing random effects that influence individual fractions. The rationale for this distinction is that systematic set-up errors are correctable, and protocols exist for minimizing their magnitude 10–12.

Uncertainties are, by definition, not correctable. However, the magnitude of the uncertainty can be determined 10–12 and its effects taken into account through the choice of margin around the ctv. Random uncertainty quantifies interfraction and intrafraction geometric effects. With the advent of image-guided radiation therapy (not considered in this study), it may be possible to view interfraction geometry changes as correctable and therefore systematic on a fraction-by-fraction basis 13.

In this study, we compared two treatment plans (one imrt and one 3D-crt) for a patient with head and neck cancer. The anatomic contours were identical, and the two dose distributions were made as comparable as possible given the different beam geometries. A Monte Carlo–based approach was used to simulate set-up error and uncertainty through a course of treatment.

## 2. METHODS

The two treatment plans were based on the objectives and constraints defined in the rtog H-0022 protocol 1. The protocol specifies dose–volume objectives for ptvs, but because ctvs were the structures of primary clinical interest for us, our analysis considers the effect of set-up error and uncertainty on the ctvs. We used Pinnacle3 software (Philips Medical Systems, Markham, Ontario) to create both treatment plans on the same set of contours.

The computed tomography (ct) volumetric data set was acquired from a patient with a T2N0 squamous-cell carcinoma of the right tonsil. The ct scans were performed using an ACQSim simulator (Philips Medical Systems) with a 3-mm slice thickness and contiguous slices and were then contoured according to the rtog H-0022 protocol guidelines by the radiation oncologist (HL). Contours were the ctv66, ctv54, and organs at risk (oars): spinal cord, brainstem, glottic larynx, right and left parotid glands, and mandible. The ct data set, including contours, was then exported to Pinnacle3 for treatment planning.

**Figure 1:**
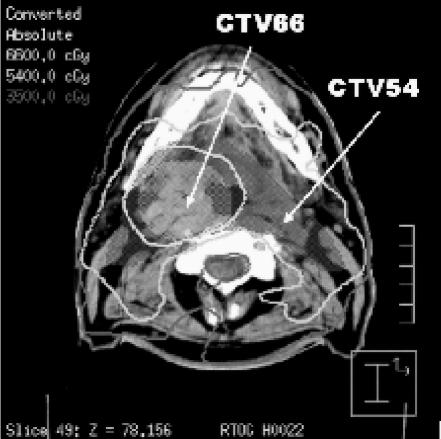
Intensity modulated radiation therapy (imrt) treatment plan showing the clinical target volumes ctv66, ctv54, and isodose lines for 66 Gy, 54 Gy, and 35 Gy.

The imrt treatment plan was developed according to the rtog protocol guidelines 1: 7-beam 6-MV “step and shoot” plan, generated using Pinnacle3 (Figure 1). The beams were co-planar and were planned for delivery at these gantry angles: 0, 51, 103, 154, 206, 257, and 309 degrees.

**Figure 2:**
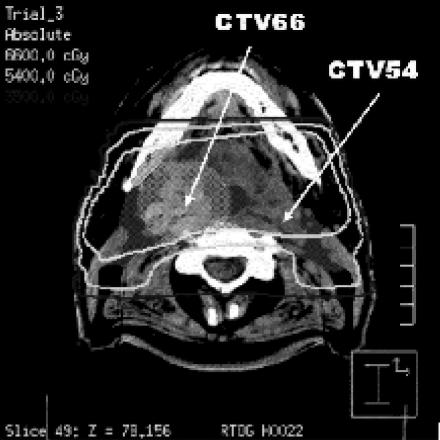
Three-dimensional conformal radiation therapy (3D-crt) treatment plan showing the clinical target volumes ctv66, ctv54, and isodose lines for 66 Gy, 54 Gy, and 35 Gy.

The 3D-crt plan (Figure 2) was developed using lateral parallel opposed fields and a matching low anterior supraclavicular field with midline spinal-cord shield. Spinal-cord blocking was introduced on the lateral fields after 19 fractions, and the right and left posterior neck areas were boosted for a further 13.2 Gy in 6 fractions using 12 MeV electrons, for a total dose of 55 Gy in 25 fractions to the ctv54. The ctv66 was treated to 66 Gy in 2.2 Gy fractions, for a total of 30 fractions. The low anterior supraclavicular field was treated to 54 Gy in 30 fractions. Thus, the ctv54 received a minimum dose of 54 Gy.

**Figure 3:**
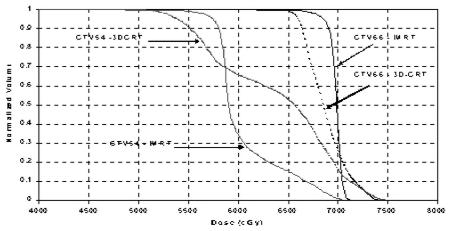
Dose–volume histograms for the clinical target volumes ctv66/54 in the static situation for the intensity modulated radiation therapy (imrt) plan (solid line) and the three-dimensional conformal radiation therapy (3D-crt) plan (dashed line).

For both plans, dose–volume histograms (dvhs) for the two ptvs and the six oars were matched as closely as possible. All were designed to satisfy H-0022 objectives and constraints. With the different beam geometries and modalities (the 3D-crt plan required posterior neck electrons), our ability to make the distributions similar was clearly limited. For our analysis, the volumes of interest were the ctvs and not the ptvs. The ptvs were used simply for planning purposes. Figure 3 shows dvhs for the ctv54 and ctv66 for both plans.

The convolution method has been shown to possibly be inappropriate for simulating geometric uncertainties when structures of interest are close to the surface 14. Our study was based on a more accurate, and more resource-intensive, Monte Carlo–based approach. Systematic errors (type B) and random uncertainties (type A) 15 were both considered. Systematic errors are known to have a greater effect on the dose distribution than random uncertainties of the same magnitude have, especially for patients with head and neck cancer 16.

In the present study, the standard deviation of the random error was fixed at 2 mm, and the standard deviation of the distribution of systematic errors for a cohort of patients was varied from 0 mm to 6 mm in increments of 1 mm. The relevant method was described in detail in a previous paper by us 17 and will be only briefly outlined here.

The first step was to use Pinnacle3 to compute dose distributions in all three orthogonal directions (anterior–posterior, lateral, superior–inferior) for shifts from −10 mm to +10 mm in increments of 1 mm. Thus, 61 pre-calculated dose distributions were obtained for each plan. Using this approach, we could account both for surface contours and for internal inhomogeneities as accurately as the algorithm permitted. For any one course, the mean displacement of the patient is represented by a single number, the systematic error. For any one fraction within that course, the displacement of the patient is chosen at random from a Gaussian distribution (σ = 2 mm) centered on the systematic error. The systematic error for a particular course was chosen at random from a Gaussian distribution intended to represent the distribution of systematic errors for a patient population. For a group of patients, such as those being simulated here, an *a priori* uncertainty exists regarding the systematic displacement of any one patient. However, once the treatment (or the computer simulation of treatment) commences, this systematic effect is fixed and may be termed an “error” for that particular patient. As previously noted, clinical protocols exist for correcting systematic effects in radiation therapy 10–12.

We used a Monte Carlo–based simulation for choosing the random displacement per fraction and the systematic error for a course within Gaussian distributions of 2 mm standard deviation for random uncertainties and 0 mm to 6 mm standard deviation for systematic errors. From the interpolation between the two closest of the 61 pre-calculated dose distributions, we used the total shift to calculate the new dose distribution. The simulations were run for 40 courses each for the two plans and for assigned systematic error. We summed 30 dose distributions, corresponding to one 30-fraction treatment course, to obtain cumulative dose distributions and dvhs for that simulated course.

We included both electron and photon contributions to the dose distribution in the dvh used to calculate the equivalent uniform dose (eud) described below. Matching of photon and electron beams is a well-known clinical issue for this type of treatment. Our approach was based on the expertise of our dosimetrists, who generated treatment plans that were consistent with our usual practice and that met with the approval of the responsible radiation oncologist.

Variability in the location of the electron fields with respect to the photon fields was not simulated. It was assumed that these abutting fields were correctly positioned with respect to each other throughout the course. Thus only patient positioning effects were considered.

The concept of the generalized eud was introduced by Niemerko 18 in the late 1990s as a means for condensing the two-dimensional dvh into one number. The eud corresponds to “the homogeneous dose distribution which produces the same surviving fraction of clonogenic cells as that obtained with an inhomogeneous dose distribution.” Specifically:


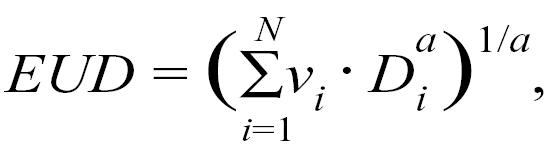


where *N* is the number of voxels, *v_i_* and *D_i_* are respectively the fractional volume and the dose at that volume, and *a* is a volume exponent that depends on the structure of interest. Through the *a* value, the eud attempts to reflect the biologic significance of a nonuniform dose distribution in the targets and the oars alike. The eud for a target will emphasize low-dose regions and de-emphasize high-dose regions.

We recently introduced the concept of eud_5%_ which is calculated from the spread of eud values calculated from the 40 (in our case) simulated treatment courses. The eud_5%_ is the eud value below which 5% of the treatment courses are estimated to fall for a target and above which 5% of the treatment courses are estimated to fall for an oar 17. The spread of eud values are Gaussian-fitted to render a statistical eud value that has a probability of 0.05. This probability-based approach makes it easy to summarize the results of multiple Monte Carlo simulations corresponding to the treatment of a cohort of patients.

## 3. RESULTS

We performed 1680 treatment-course simulations (840 for imrt and 840 for 3D-crt) in the three orthogonal directions for seven distributions of systematic error ranging from 0 mm to 6 mm standard deviation.

Figures 1 and 2 show the dose distributions of the imrt and 3D-crt plans respectively. Superior conformality of the dose to the tumour for the imrt plan is clear from Figure 1.

Figure 3 shows the dvhs of the ctv54 and ctv66 for both the imrt plan and the 3D-crt plan.

**Figure 4:**
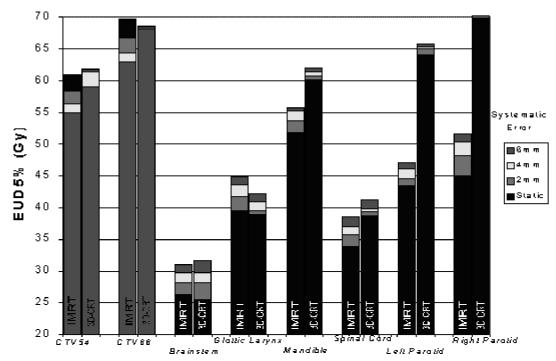
The 5% equivalent uniform dose (eud_5%_) to the two targets (clinical target volumes ctv54 and ctv66) and six organs at risk (oars) in the static situation (black) and in presence of systematic set-up error. Random uncertainty was fixed at 2 mm standard deviation. The eud_5%_ is the eud value below which 5% of the treatment courses are estimated to fall for a target and above which 5% of the treatment courses are estimated to fall for an oar.

Figure 4 shows the eud_5%_ of the two targets and the six oars in the static situation and in the presence of set-up error and uncertainty. The eud_5%_ on the histograms corresponds to the average eud_5%_ over the three orthogonal directions studied in the simulations. For clarity, we present the results for the static situation and for the 2 mm, 4 mm, and 6 mm standard deviations only.

## 4. DISCUSSION

Evaluation of a treatment plan using dvh data alone may be inadequate. On Figure 3, the dvhs are seen to cross, and concluding which treatment technique gives the better dose distribution is difficult. Examining the eud together with the dvh and the 3D dose distribution assists in identifying the optimum plan.

In the static situation, the eud_5%_ to the ctv66 in the imrt plan is slightly higher than the eud_5%_ to the ctv66 in the 3D-crt plan, although both have the same isocentre dose. Interestingly, the eud_5%_ to the ctv54 is lower in the imrt plan because of the irregular shape of the ctv54 and the difference in the geometric configuration of the beams for the two plans. The eud_5%_ to the brainstem and the glottic larynx show no significant differences. The real benefits of the imrt plan over the 3D-crt plan, quantified in terms of eud, accrue to the spinal cord (12.6% reduction in eud_5%_), the mandible (13.8% reduction in eud_5%_), the left parotid (32.2% reduction in eud_5%_), and the right parotid (35.5% reduction in eud_5%_). Parotid sparing is the principal objective of the rtog protocol and is clearly achieved with this imrt plan in the static situation.

Figure 4 also shows the variation in the eud_5%_ for the eight structures when set-up error and uncertainty are incorporated into the treatment plans. As set-up error increases from 0 mm to 6 mm, the eud_5%_ to the ctv66 and ctv54 decline and the eud_5%_ to the oars rise in both plans, but by different amounts. For example, the eud_5%_ to ctv66 in the static situation for the imrt plan is 69.8 Gy. In the presence of a 2-mm standard deviation in systematic error, this eud_5%_ drops to 66.8 Gy. Set-up error on ctv54 for 3D-crt can be seen to have little effect. Indeed, the eud is reduced by less than 1% for a 6-mm systematic setup error.

From Figure 4, we also observe that sensitivity to set-up error and uncertainty is greater in the imrt plan than in the 3D-crt plan for the targets and all six oars. However, when a patient is treated with 3D-crt, the target will likely receive an adequate dose even in presence of set-up uncertainties. Particularly, lateral shifts on the 3D-crt will have very little impact on the ctv66/54 because the treatment uses parallel opposed beams. However, imrt retains its overall superiority with respect to the difference between doses to the target and the oars. In particular, even in the presence of large set-up error, the eud_5%_ values in the imrt plan are 27% lower for the right parotid and 29% lower for the left parotid than they are in the 3D-crt plan. Thus set-up error, within the range examined, does not compromise the parotid gland–sparing capability of imrt.

The eud_5%_ equation used in this study does not take into account dose-per-fraction effects. The only structure considered to be of relevance in this regard is the spinal cord, which, in the 3D-crt approach, is blocked after 19 fractions. To account for this factor, we calculated the equivalent uniform biologically effective dose (eubed) 19 for the spinal cord,


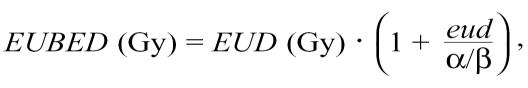


where *eud* is the total eud received by the spinal cord in grays, *eud* is the eud per fraction in grays, and the ratio α/β (the dose at which the linear and quadratic components of the radiation damage are equal) is 3 Gy for the spinal cord 20. This calculation is an attempt to normalize the larger fraction size to the spinal cord in the 3D-crt plan with respect to the equivalent in the imrt plan in terms of biologic effect. The eubed for the spinal cord in the static situation was 45.6 Gy (eud = 33.8 Gy) for the imrt plan and 65.0 Gy (eud = 38.7 Gy) for the 3D-crt plan. Thus, for the particular case of the spinal cord, which is blocked after 19 fractions in the 3D-crt plan, increased superiority in the imrt plan is demonstrated when fraction size is taken into account. Adjusting for the different fraction size, the eubed to the spinal cord is 28% lower in the imrt plan.

Set-up error and uncertainty can lower the dose to the targets while the dose to the oars increases. However, the original eud of the target can be restored simply by increasing the isocentre dose by the fractional amount of eud lost in the presence of set-up uncertainty. This change will, of course, increase the dose to the oars. However, based on our results, even with an increase in the isocentre dose necessary to restore the target eud, the euds to the oars—and especially the parotid glands, the main structures of interest in this study—will remain well below the dose reached in the 3D-crt plan. An opportunity therefore exists either to increase the target dose to restore the euds of the oars to their static values or to maintain the target eud at its static value and to provide greater protection to the oars. The eud formalism lends itself to this sort of approach because euds scale linearly with dose.

## 5. CONCLUSION

We have shown that set-up error and uncertainty in the clinical situation have an impact on the euds received by the targets and by the critical structures in both imrt and 3D-crt head and neck treatment plans. Even though the effect is more noticeable with imrt, the imrt treatment technique retains its superiority over 3D-crt in presence of set-up error and uncertainty. After accounting for the effects of fraction size on the eud to the spinal cord, that structure is seen to receive additional sparing because of the lower dose per fraction of imrt. Finally, the eud approach lends itself to compensation for patient positioning effects, if required. All euds scale to the dose at any point. Thus, the effect of an increase (or decrease) of the isocentre dose can be immediately calculated for the target and oar euds, permitting an estimate of the probability that a treatment course will achieve given euds.
